# Contrasting Determinants of Mutation Rates in Germline and Soma

**DOI:** 10.1534/genetics.117.1114

**Published:** 2017-07-21

**Authors:** Chen Chen, Hongjian Qi, Yufeng Shen, Joseph Pickrell, Molly Przeworski

**Affiliations:** * Department of Biological Sciences, Columbia University, New York, New York 10025; † New York Genome Center, New York, New York 10013; ‡ Department of Systems Biology, Columbia University Medical Center, New York, New York 10032; § Department of Applied Physics and Applied Mathematics, Columbia University, New York, New York 10025; ** Department of Biomedical Informatics, Columbia University, New York, New York 10025

**Keywords:** human, mutation rate, germline mutations, somatic mutations, strand asymmetry

## Abstract

Recent studies of somatic and germline mutations have led to the identification of a number of factors that influence point mutation rates, including CpG methylation, expression levels, replication timing, and GC content. Intriguingly, some of the effects appear to differ between soma and germline: in particular, whereas mutation rates have been reported to decrease with expression levels in tumors, no clear effect has been detected in the germline. Distinct approaches were taken to analyze the data, however, so it is hard to know whether these apparent differences are real. To enable a cleaner comparison, we considered a statistical model in which the mutation rate of a coding region is predicted by GC content, expression levels, replication timing, and two histone repressive marks. We applied this model to both a set of germline mutations identified in exomes and to exonic somatic mutations in four types of tumors. Most determinants of mutations are shared: notably, we detected an effect of expression levels on both germline and somatic mutation rates. Moreover, in all tissues considered, higher expression levels are associated with greater strand asymmetry of mutations. However, mutation rates increase with expression levels in testis (and, more tentatively, in ovary), whereas they decrease with expression levels in somatic tissues. This contrast points to differences in damage or repair rates during transcription in soma and germline.

## Abstract (precis)

A number of genomic features influence regional mutation rates in germline and soma. To examine if some factors behave differently in the two tissue...

GERMLINE mutations are the source of all heritable variation, including in disease susceptibility, and it is increasingly clear that somatic mutations also play important roles in human diseases, notably cancers (Muller 1927; Stratton *et al.* 2009). Understanding the rate and mechanisms by which mutations occur is therefore of interest to both evolutionary biologists and to human geneticists aiming to identify the underlying causes of genetic diseases (Shendure and Akey 2015; Gao *et al.* 2016). In particular, an accurate estimate of the local mutation rate is key to testing for an excess of disease mutations in specific genes among cases (Lawrence *et al.* 2013; Samocha *et al.* 2014). Characterization of the variation in mutation rate along the genome can also yield important insights into DNA damage and repair mechanisms (Stratton 2011; Ségurel *et al.* 2014).

Until recently, our understanding of germline point mutations came mainly from analysis of diversity along the genome or divergence among species (Green *et al.* 2003; Webster *et al.* 2004; Polak and Arndt 2008; Hodgkinson and Eyre-Walker 2011; Park *et al.* 2012). In the past several years, analyses have also been based on resequencing exomes or whole genomes from blood samples of human pedigrees and calling germline variants as those present in the offspring but absent in the child (reviewed in Campbell and Eichler 2013; Ségurel *et al.* 2014; Shendure and Akey 2015). This approach is more direct than analyzing divergence data, and presents the advantage of being almost unaffected by selection, but the analysis is technically challenging and, with current study designs, some mutations may be missed, notably those that occur in the early postzygotic divisions (Harland *et al.* 2016; Moorjani *et al.* 2016; Rahbari *et al.* 2016).

Our knowledge of somatic point mutations, in turn, relies primarily on resequencing tumors. In these analyses, mutation calls are made by sequencing tumor and noncancerous tissue pairs from the same individual, and then excluding the variants shared between the two tissues (as the shared mutations are likely to be germline). Because, in this approach, a large population of cells is sequenced, the mutations identified tend to predate the tumorigenesis, and thus are mostly somatic mutations that occurred in normal tissues (see, *e.g.*, Alexandrov *et al.* 2015; Martincorena *et al.* 2015).

Studies of both germline and soma reveal that the point mutation rate varies across the genome, from the scale of a single base pair to much larger scales (Hodgkinson and Eyre-Walker 2011; Hodgkinson *et al.* 2012; Ségurel *et al.* 2014). At the single base pair level, the largest source of variation in germline mutation rate is the identity of the adjacent base pairs (Hwang and Green 2004; Hodgkinson and Eyre-Walker 2011). Notably, the mutation rate of CpG transitions (henceforth CpG Ti) is an order of magnitude higher than other mutation types (*e.g.*, Kong *et al.* 2012). Most CpG dinucleotides are methylated in the human genome; when the methylated cytosine undergoes spontaneous deamination to thymine and is not corrected by the time of replication, the damage leads to a mutation. Among other types of sites, rates of mutation vary by two- to threefold (Kong *et al.* 2012). In the soma, the mutation rate at CpG sites is also elevated, although the extent of the increase differs across tumor types (Lee *et al.* 2010; Pleasance *et al.* 2010a,b). More generally, tumors vary in their mutation spectrum: analyses of mutations and their two neighboring base pairs (*i.e.*, considering 96 mutation types) point to enrichment of distinct mutational signatures for different types of cancers, a subset of which have been shown to reflect particular mutagens or differences in the efficiency of repair (Alexandrov *et al.* 2013).

Over a larger scale of megabases, germline mutation rates have been associated with a number of additional factors, including transcription level (in testis), replication timing (in lymphoblastoid cell lines), chromatin states (both in lymphoblastoid cells and in ovary), meiotic crossover rates, and GC content (Hodgkinson and Eyre-Walker 2011; Michaelson *et al.* 2012; Park *et al.* 2012; Francioli *et al.* 2015; Besenbacher *et al.* 2016; Goldmann *et al.* 2016). Somatic mutation rates have also been associated with replication timing (in Hela cell lines) and with average transcription levels across 91 cell lines in Cancer Cell Line Encyclopedia (Lawrence *et al.* 2013).

In many cases, little is known about the mechanistic basis for the association of a given factor with mutation rates. However, the association of somatic mutation rates with transcription levels appears to be a byproduct of transcription-coupled repair (TCR), a subpathway of nucleotide excision repair (NER) (Hanawalt and Spivak 2008; Nouspikel 2009). NER is a versatile repair pathway that senses lesion-causing distortions to DNA structure and excises the lesion for repair. Another subpathway of NER, global genome repair (GGR), can repair lesions on both transcribed strand (TS) and nontranscribed strand (NTS), including transcribed regions as well as transcriptionally silent ones. In contrast, TCR operates only within transcribed regions, triggered by lesions on the TS, which it repairs off the NTS. This mechanism gives rise to a mutational strand asymmetry, as well as a compositional asymmetry between strands. For example, TCR leads to more A–G mutations (henceforth A > G) on the NTS than TS; acting over long periods of time, this phenomenon generates an excess of G over A (and T over C) on the NTS (Green *et al.* 2003; McVicker and Green 2010). Such mutational strand asymmetry has been found in both germline and soma (Green *et al.* 2003; Polak and Arndt 2008; Rubin and Green 2009; Lawrence *et al.* 2013; Francioli *et al.* 2015; Martincorena *et al.* 2015).

While many of the same determinants appear to play important roles in both germline and soma, there are hints of differences as well. For instance, studies of preneoplastic somatic mutations indicate that, over a 100 kb scale, the mutation rates in somatic tissues decrease with expression levels, and increase with later replication timing (Lawrence *et al.* 2013). Similarly, two studies that focused on somatic mutations in noncancerous somatic tissues, normal eyelid tissue, and neurons, found mutations to be enriched in regions of low expression and repressed chromatin (Lodato *et al.* 2015; Martincorena *et al.* 2015). A similar effect of replication timing was identified in studies of germline mutation (Stamatoyannopoulos *et al.* 2009; Francioli *et al.* 2015; Besenbacher *et al.* 2016; Carlson *et al.* 2017). However, the effect of expression levels on germline mutation rates remains unclear: one study reported increased divergence between humans and macaques with greater germline expression (Park *et al.* 2012), but others found no discernable effect of expression levels on mutation rates (Green *et al.* 2003; Webster *et al.* 2004; Polak and Arndt 2008; Hodgkinson and Eyre-Walker 2011; Francioli *et al.* 2015). This difference between germline and soma is particularly puzzling in light of the observation that the strand asymmetry of mutation rates between TS and NTS is seen in the germline as well as the soma (McVicker and Green 2010; Pleasance *et al.* 2010a,b; Lawrence *et al.* 2013). Together, these observations suggest that the determinants of mutation rates may differ between germline and soma, raising the more general possibility that the damage rate or the repair efficacy differs among cell types (Lynch 2010).

A limitation, however, is that studies have used different statistical approaches, rendering the comparison hard to interpret. As an illustration, whereas some studies binned the genome into windows of 100 kb (*e.g.*, Lawrence *et al.* 2013) or 1 Mb regions (*e.g.*, Polak *et al.* 2015), other studies have compared the mean mutation rate in transcribed regions and nontranscribed regions or in genes grouped by expression levels (Hodgkinson and Eyre-Walker 2011; Francioli *et al.* 2015; Lodato *et al.* 2015). Studies of somatic mutation also vary in whether they group different tissues or distinguish among them (*e.g.*, Pleasance *et al.* 2010b; Lawrence *et al.* 2013). An additional limitation of earlier studies of germline mutation is that, by necessity, they relied on human–chimpanzee divergence as a proxy for *de novo* mutation rates (Green *et al.* 2003; Webster *et al.* 2004; Hodgkinson and Eyre-Walker 2011), even though divergence reflects not only the mutation process but also effects of natural selection in the human–chimpanzee ancestor and biased gene conversion (Duret and Galtier 2009; McVicker *et al.* 2009).

To our knowledge, only one study has used a uniform approach to study germline and soma. Their findings point to possible differences in their determinants: for instance, the histone mark H3K9me3 accounts for >40% of mutation rate variation at 100 kb in tumors, when a much weaker association is seen in the germline (Schuster-Böckler and Lehner 2012). This analysis relied on pairwise correlations, however, and therefore the results may be confounded by other factors that are correlated to the histone marks and differ between tissues. Moreover, to our knowledge, there has been no parallel treatment of strand asymmetry in germline and soma.

To overcome these limitations, we built a multivariable regression model, in which the mutation rates of CpG Ti and other types of mutations in a coding region are predicted by GC content, expression levels, replication timing and two histone repressive marks. To this end, we used the expression levels, replication timing and histone marker levels of matched cell types, when available. We applied the model to a large set of germline point mutations identified in exomes from recently published studies on developmental disorders and to somatic point mutations in exomes found in four types of tumors and reported by the Cancer Genome Atlas (see *Materials and Methods*). In addition, we considered the mutational strand asymmetry in the two sets of data.

## Materials and Methods

### Datasets

To study germline mutations, we relied on *de novo* mutation calls made from 8681 trios surveyed by exome sequencing. We combined results from two main sources: studies of neurodevelopmental disorders (NDD), which considered 5542 cases and 1911 controls (unaffecteds), and studies of congenital heart defect (CHD), conducted by the Pediatric Cardiac Genomics Consortium, which included 1228 trios. The NDD cases include 3953 cases of Autism Spectrum Disorder (ASD), 1133 cases of deciphering developmental disorders (DDD), 264 cases of epileptic encephalopathies (EE), and 192 cases of intellectual disability (ID). All these studies applied similar capture and sequencing methods, and most samples were at >20× coverage (see Table 1).

**Table 1:** Summary of germline datasets

Dataset	Trios	References	Capture	Sequencing
ASD	3953	De Rubeis *et al.* (2014); Iossifov *et al.* (2014)	Exome	Illumina and SOLiD
Simons simplex collection, unaffected	1911	Iossifov *et al.* (2014)	Exome	Illumina
CHD	1213	Homsy *et al.* (2015)	Exome	Illumina
DDD	1133	The Deciphering Developmental Disorders Study (2015)	Exome	Illumina
EE	264	Epi4K Consortium *et al.* (2013)	Exome	Illumina
ID	192	de Ligt *et al.* (2012); Rauch *et al.* (2012); Hamdan *et al.* (2014)	Exome	Illumina

We tested for an effect of the study, which could potentially arise from differences in design or analysis pipeline, by adding a categorical variable (by an analogous approach to the one described below to test for differences among tissues). We found a marginally significant interaction between the study and the expression level in testis (our proxy for expression levels in the germline), driven by one study (CHD cases; Homsy *et al.* 2015), as well as for interactions between the studies and the effects of H3K9me3 and GC content, driven by two small studies (EE and ID) (see Supplemental Material, Figure S1 in File S1). Given these minor differences, and in order to increase our power, we combined all the germline mutation datasets in what follows (see Table S1 for list of mutations).

To examine determinants of mutation rates in somatic tissues, we downloaded somatic mutation calls identified in four types of cancer from the Cancer Genome Atlas (TCGA) portal (in July 2015): breast invasive carcinoma (BRCA), cervical squamous cell carcinoma and endocervical adenocarcinoma (CESC), brain lower grade glioma (LGG), and liver hepatocellular carcinoma (LIHC). The numbers of samples are listed below (Table 2). In all cases, both noncancerous and tumor tissues of patients were sampled, and the exomes were sequenced using an Illumina platform. In the studies, mutation calls shared by the normal and tumor samples were removed (on the presumption that they are germline). What remains are somatic mutations found at high enough frequency to be seen in a large population of cells, which are therefore likely to predate the tumorigenesis, *i.e.*, that occurred in the preneoplastic tissues (Martincorena *et al.* 2015).

**Table 2:** Sizes of TCGA datasets

Dataset	Sample size
BRCA	904
CESC	181
LGG	502
LIHC	171

For each type of cancer with more than one mutation annotation file available in the TCGA data portal, we selected the file that included the largest number of patient samples. We removed the ∼7.6% of samples that had an unusually large number of mutations per sample (*P* < 0.05 by Tukey’s test), because they are likely to reflect loss of some aspect of the DNA mismatch repair, and hence their mutational mechanisms likely differ (Supek and Lehner 2015).

### Possible determinants of mutation rates

We considered the main factors previously reported to be significantly correlated with mutation rates, namely expression levels, replication timing, GC content, and histone modification levels. To quantify expression levels, we relied on gene expression data (measured as RPKM) from the Genotype-Tissue Expression (GTEx) for breast, uterus, brain cortex, and liver tissues. We also used GTEx expression data from testis and ovary tissues, as proxies for germline cells.

The effect of the replication timing on somatic mutation rates was argued to be cell-type specific (Supek and Lehner 2015). We therefore relied on Repli-Seq measurements (provided per base pair) in ENCODE cell lines that match the four types of cancer, namely MCF-7 (breast cancer), Hela-S3 (cervical cancer), SK-N-SH (neuroblastoma), and HepG2 (liver hepatocellular carcinoma) cell lines. These measurements were obtained from the University of California, Santa Cruz (UCSC) Genome Browser. In all cases, the replication timing reported is a smooth measure of the relative enrichment of early *vs.* late S-phase nascent strands, with high values indicating early replication. For each gene, we computed the average replication timing by taking the mean value of the data points that overlap with gene start-to-end coordinates in UCSC Refseq gene database. For genes with multiple transcripts, we took the union of all exons in all transcripts. For germline mutations, there are no data for the appropriate cell types, so we used replicating timing estimates for lymphoblastoid cell lines (LCL) (provided in 10 kb windows) (Koren *et al.* 2012). We also tried using replication timing data from three somatic tissues instead; the replication timing data are highly correlated among the tissues, and, accordingly, the effects of mutation were estimated to be very similar (see Figure S2 in File S1).

In addition, we considered the effects of chromatin marks that had been shown to correlate individually with somatic and germline mutation rates (Schuster-Böckler and Lehner 2012; Carlson *et al.* 2017): specifically, histone modification H3K9me3 and H3K27me3, two repressive marks associated with constitutively and facultatively repressed genes, respectively. Levels of these marks were downloaded from roadmap epigenomics data browser (December 2015, hg19) and converted to gene-based histone modification levels by averaging across the gene. We used the histone modification levels of adult ovary, breast myoepithelial cells, brain hippocampus, and adult liver as proxies for germline, breast, brain, and liver, respectively. In the following regression analysis, we considered only three of four somatic tissues, as we could not obtain histone modification data for CESC. Finally, we computed exonic GC content as the fraction of G or C residues in the union of exons in all isoforms of a given gene.

Germline mutation studies relied on the UCSC Refseq gene annotation, whereas TCGA uses GENECODE annotation, which contains more transcripts (Larsson *et al.* 2005; Zhao and Zhang 2015). To make the comparison cleaner, we focused on exonic regions considered in both types of studies by using gene and exon coordinates of Refseq database in build hg19 from the UCSC genome browser.

### Statistical model

Our main goal was to investigate possible relationships between mutation rates and gene expression levels, while controlling for replication timing, GC content and some histone modification levels. Because our mutation counts are overdispersed, with greater variance than mean, we used a negative binomial regression model (instead of, *e.g.*, a Poisson regression model). Specifically, for every protein-coding gene, we counted the number of CpG Ti or other types of mutations in the coding exons of a gene and treated it as an outcome of a sequence of independent Bernoulli trials with probability ${\text{λ}}_{\text{i}}$, where ${\text{λ}}_{\text{i}}$ is the probability of a mutation occurring in gene i.

Transitions at CpG sites are thought to occur primarily due to spontaneous deamination at methylated cytosines, a distinct mutational source, and thus their determinants may be distinct from other mutation types (reviewed in Ségurel *et al.* 2014). However, within CpG islands, most CpGs are hypomethylated (Takai and Jones 2002). To focus on a more homogeneous set of methylated CpGs, we therefore excluded CpG islands from the analyses of CpG Ti. CpG island annotations were downloaded from UCSC browser (track: CpG Islands). In one analysis (Figure 2), we included the average level of CpG methylation in each gene in our model, as assayed by bisulfate sequencing in ovary, sperm, breast myoepithelial cells, brain hippocampus cells, and adult liver cells.

We considered gene expression levels measured in RPKM (X_1_), replication timing (X_2_), mean histone modification levels (H3K9me3 as X_3_ and H3K27me3 as X_4_), and GC content (X_5_) as predictors. We also included L, the total number of CpG sites (when considering CpG Ti) or all nucleotides (when considering all other types of mutations) in the exons of the given gene, as an exposure variable, to account for the variation in gene length. The logarithm of ${\text{λ}}_{\text{i}}$ is then modeled as a linear combination of these features scores:

$$\log\left(\lambda_{\text{i}}\right)=\beta_{0}+\overset{5}{\underset{j=1}{\sum }}\beta_{\text{j}}{\text{X}}_{\text{ij}}+\log\left(\text{L}\right)+\varepsilon$$

We used R function glm.nb to estimate the coefficients, where $\beta_{0}$ is an intercept term, $\beta_{\text{j}}\;$is the effect size of feature j, and ${\text{X}}_{\text{ij}}$ is the score for feature j in gene i. In order to make the effect sizes of different features comparable within a model, we normalized all the predictor variables to have a mean of 0 and a SD of 1. The gene expression levels measured in RPKM originally range from 0 to a few hundred thousand. As is standard (*e.g.*, Green *et al.* 2003; Francioli *et al.* 2015), we added half of the smallest nonzero value in the corresponding expression data sets and then log-transformed the expression level before normalization.

We note that, in this model, we are considering possible effects one at a time. Including interaction terms affects the estimates and significance levels but changes none of the qualitative results, with the exception of results for H3K27me3, which become less significant (see Figure S3 in File S1).

To examine whether the predictors have significantly different effects across tissues, we combined the models into one by including a categorical variable C for the tissue type (see Figure 3). In this approach:

$$\begin{array}{l} \mathrm{C=1}\;\text{for}\;\text{somatic}\;\text{tissues,}\;C=0\;\text{for}\;\text{germline;}\\ \log\left(\lambda_{\text{ij}}\right)=\beta_{0}+\overset{5}{\underset{j=1}{\sum }}\beta_{\text{j}}{\text{X}}_{\text{ij}}+\text{C}\left(\beta_{6}+\overset{11}{\underset{j=7}{\sum }}\beta_{\text{j}}{\text{X}}_{\text{ij}}\right)+\text{log}(\text{L})+\varepsilon \end{array}$$

where X_1_, X_2_, X_3_, X_4_ and X_5_ are the same genomic or epigenomic features as in the separate model, *β*_1,_ *β*_2,_ *β*_3_, *β*_4_, *β*_5_ are the effect sizes of features X_1_ to X_5_ for testis, and *β*_7_, *β*_8_, *β*_9_, *β*_10_, and *β*_11_ are the differences of effect size in the somatic tissue of features X_1_ to X_5_ compared to those in testis. We used the R function glm.nb to estimate the coefficients.

Similarly, in order to ask whether effects differ between CpG Ti and other type of mutations in the same tissue, we included a binary variable C for the two mutation types (see Figure S4 in File S1) where:

$$\begin{array}{l} \mathrm{C=}\text{1}\;\text{for}\;\text{CpG}\;\text{Ti},\;C=0\;\text{for}\;\text{all}\;\text{other}\;\text{mutations;}\\ \log\left(\lambda_{\text{ij}}\right)=\beta_{0}+\overset{5}{\underset{j=1}{\sum }}\beta_{\text{j}}{\text{X}}_{\text{ij}}+\text{C}\left(\beta_{6}+\overset{11}{\underset{j=7}{\sum }}\beta_{\text{j}}{\text{X}}_{\text{ij}}\right)+\text{log}(\text{L})+\varepsilon \end{array}$$

All variables are set up the same way as in the combined model described previously, except for that *β*_7_, *β*_8_, *β*_9_, *β*_10_, and *β*_11_ are now the differences of the effect sizes for CpG Ti compared to those for all other mutation types.

### Mutation spectrum and strand asymmetry analysis

We annotated the direction of transcription using the UCSC CCDS track and filtered out genes that are transcribed off both strands (1.7% of genes in Refseq), which left ∼19,000 genes to consider. This annotation allowed us to classify mutations into six types of mutation (A > C, A > G, A > T, G > A, G > C, G > T) on either TS or NTS. There are thus 12 possible changes (each of the six on both strands). We then calculated the mutation rate of any given type on NTS and TS separately, by considering the number of corresponding mutations in the combined data sets, divided by the total number of nucleotides that could give rise to such a mutation in the exons. To obtain the confidence intervals on the mutation rates (reported in Figure 4, Figure 5, and Figure S5 in File S1) as well as for the mutation asymmetry ratio (Figure 5 and Figure S5 in File S1), we used bootstrap resampling. Specifically, we created 100 samples of the same size as the original sample, by drawing randomly from the original sample with replacement, and estimated the 95% CI from those 100 samples.

We tested for strand asymmetry by a Chi-squared test. Because A > G strand asymmetry shows the greatest asymmetry (Green *et al.* 2003), and is the only mutation type that we found to be significant (and in the same direction) in all tissues (Figure 4), we focused primarily on this type, though we also considered A > T mutational patterns (see Figure S5 in File S1). To test if the extent of strand asymmetry changes with transcription levels, we grouped genes into expression level quantiles and calculated A > G strand asymmetry. Our measure of strand asymmetry is the ratio of the mutation rate on NTS to that on TS.

**Figure 1:**
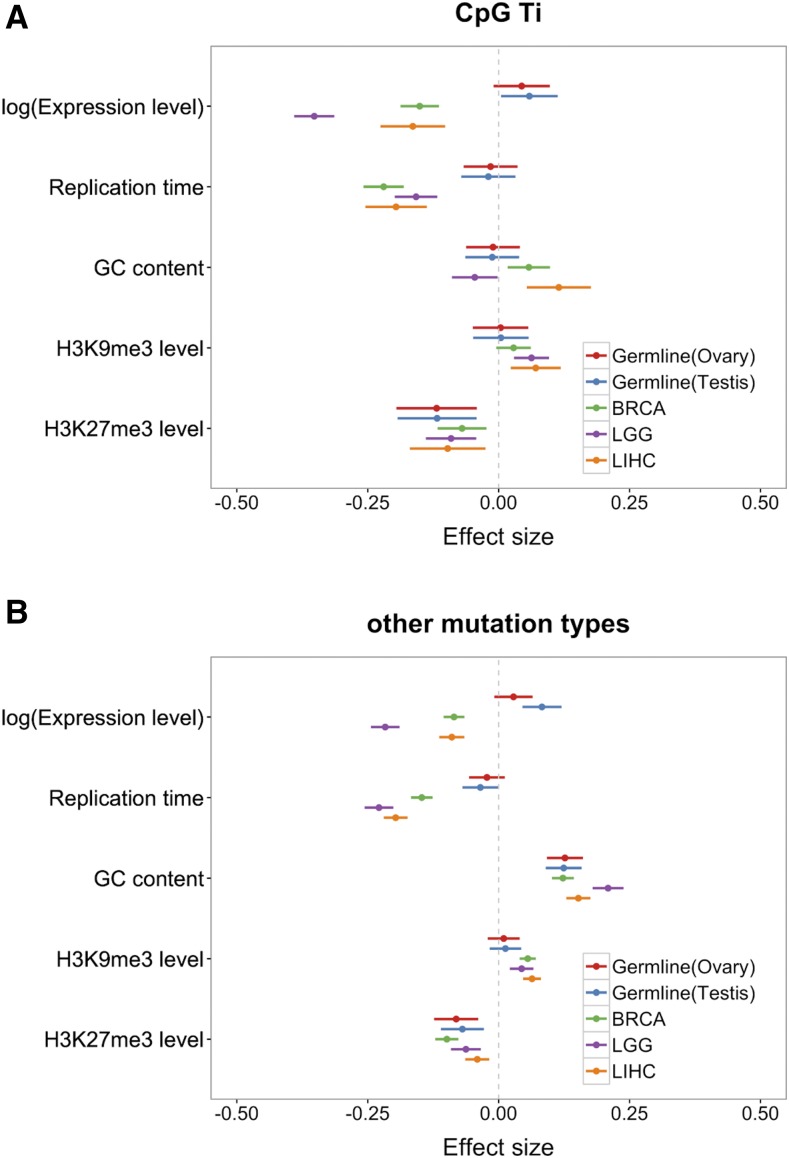
Coefficients of multivariable binomial regression model fit to germline and somatic mutation data. (A) Results for CpG Ti, (B) results for other mutation types. Red, blue and green, purple and orange bars represent the 95% CI for the estimate of the regression coefficient in germline data set using ovary expression and testis expression, BRCA, LGG, and LIHC data sets, respectively. For all replication timing data, a higher value means earlier.

We also considered the A > G mutation rates on NTS and TS separately (in Figure 6). Here, the number of A > G mutations on NTS and TS for gene *i* is treated as an outcome of a sequence of independent Bernoulli trials with probability $\lambda_{i}$ as the response variable in the model below.

$$\log\left(\lambda_{\text{i}}\right)=\beta_{0}+\overset{5}{\underset{j=1}{\sum }}\beta_{\text{j}}{\text{X}}_{\text{ij}}+\log\left(\text{L}\right)$$

where *L* is the corresponding number of As on the NTS or TS of a gene and other predictor variables are set up the same way as in the separate model in Figure 1. We applied the same analysis to A > T mutations, for which we detected significant asymmetry in the same direction in all types of tissues except for LGG (Figure S6 in File S1).

### Data availability

Germline mutations are provided in Table S1. TCGA somatic mutations can be downloaded from GDC data portal (https://gdc-portal.nci.nih.gov/). The replication timing data of LCL and other tissues are available from Koren *et al.* (2012) and the ENCODE website (https://www.encodeproject.org/search/?type=Experiment&assay_title=Repli-seq), respectively. The histone modification data can be freely accessed from the Epigenome Roadmap website (http://www.roadmapepigenomics.org/data/tables/all). CpG methylation data were downloaded from GEO (https://www.ncbi.nlm.nih.gov/geo/), with accession number GSM1010980 for ovary, GSM1127119 for sperm, GSM11217054 for breast myoepithelial cells, GSM1112838 for brain hippocampus cells, GSM916049 for adult liver cells, and GSM429321 for ESC cells.

## Results

### Variation among germline and somatic tissues

We began by applying our multivariable regression model (see *Materials and Methods*) to compare the determinants of mutation rates per gene between the two germline tissues, and among the three somatic tissues (Figure 1). Results for germline mutations are very similar using testis or ovary expression profiles.

**Figure 2:**
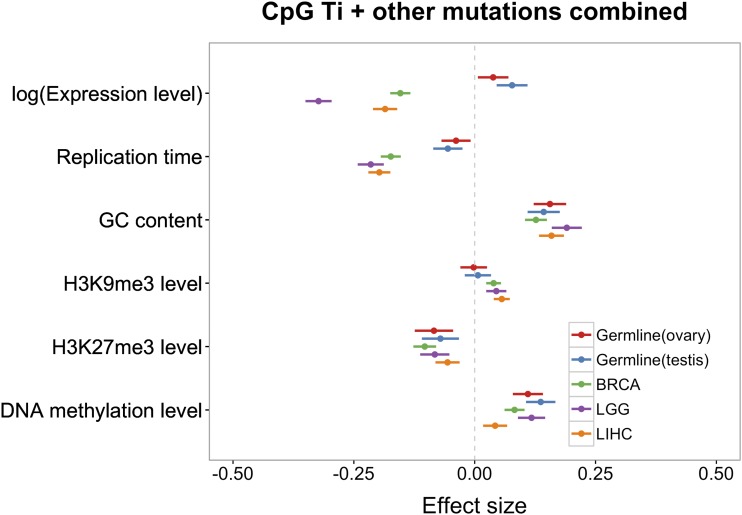
Coefficients of multivariable binomial regression model with DNA methylation levels added as a predictor, fit to germline and somatic mutation data. Red, blue and green, purple and orange bars represent the 95% CI for the estimate of the regression coefficient in germline data set using ovary expression and testis expression, BRCA, LGG, and LIHC data sets, respectively. For all replication timing data, a higher value means earlier.

Notably, in both testis and ovary, we found little effect of replication timing on germline mutation rates, other than a marginally significant negative effect for mutations other than CpG Ti (*P* = 0.046, using testis expression data). An association of replication timing had been previously reported for (imperfect) proxies of *de novo* mutation rates (Stamatoyannopoulos *et al.* 2009), suggesting that our inconclusive findings may reflect lack of power. Indeed, if we combine all mutation types within a coding region, and add CpG methylation levels within the gene as a covariate, the effect of replication timing is more readily apparent (Figure 2; *P* = 3.3 × 10^−4^ using testes expression data, and *P* = 0.01 using ovary expression data).

We also detected a significant increase of germline mutation rates with expression levels for both CpG Ti and other mutation types (Figure 1; see also Figure S2 in File S1 for similar results with replication timing for different tissues), in contrast to a previous study using *de novo* mutations (Francioli *et al.* 2015) and most previous studies of divergence. One difference with the previous analysis of *de novo* mutations is that we rely on exonic mutation data and focus on the unit of a gene, whereas they considered whole genome data, dividing it up into 100 kb windows.

In our analysis, the effect of expression levels is most clearly seen using testis expression (*e.g.*, in Figure 2, *P* = 2.1 × 10^−6^) rather than ovary expression (*P* = 0.02), possibly due to the fact that over three-quarters of germline mutations are of male origin (Kong *et al.* 2012; Goldmann *et al.* 2016; Rahbari *et al.* 2016). Alternatively, the ovary expression profile may be a poorer proxy for female germ cells than the testis expression profile is for male germ cells. In any case, henceforth, we use testis expression profile for analysis of the germline mutation rates, unless otherwise noted.

We note that our analysis of germline mutation relies on *de novo* mutation calls made in exome studies of blood samples from six sets, including five cases and unaffected controls (see Table 1). A previous study reported that in one set of cases, individuals with CHD, there is an increased number of putatively damaging mutations in the genes most highly expressed in the developing heart and brain (Homsy *et al.* 2015). Since the mutations are thought to be germline mutations (rather than somatic mutations), this association cannot be causal, instead reflecting an enrichment of damaging mutations in important heart developmental genes in CHD patients. To evaluate whether our findings of increased mutation rates with germline expression levels could be driven by a similar ascertainment bias, we excluded the CHD set and obtained the same results (see Figure S7 in File S1). We also reran the analysis, comparing the effects in the five cases compared to the controls; none of the qualitative results differed, though, as expected from the smaller size of the control sets, the estimated effect sizes were more uncertain (see Figure S8 in File S1). Thus, our results suggest that the increase in mutation rates with expression levels in testes is not a result of focusing primarily on cases.

Germline mutation rates involving CpG Ti and other types are negatively associated with H3K27me3 levels (Figure 1). We also found that, other than for CpG Ti, germline mutation rates increase with the GC content of a gene. This observation is consistent with previous findings of a high rate of GC to AT mutations relative to other types (*e.g.*, Kong *et al.* 2012). In addition, it is thought that misincorporated bases during DNA replication in AT rich regions are more easily accessible, and thus more easily repaired than GC rich regions (Petruska and Goodman 1985; Bloom *et al.* 1994). Indeed, considering only AT sites, mutation rates increase in regions of higher GC (see Figure S9 in File S1), indicating that there is an effect of the GC content of nearby sites, not only of the higher mutation rate of GC sites themselves.

Among somatic tissues, the effects of mutation rate predictors are also concordant. Notably, mutation rates decrease with expression levels in all three tissues, though the magnitudes of the effects differ. This finding is consistent with previous studies and thought to be a result of TCR (Lawrence *et al.* 2013). Intriguingly, in a model comparing the effects on CpG Ti and other mutation types directly, in all three somatic tissues, the effect of expression levels on mutation rates is most pronounced for CpG Ti (see Figure S4 in File S1). This finding suggests that damage or repair of CpG Ti is tightly coupled to transcription.

In all three somatic tissues, a later replication timing, a decrease in H3K27me3 levels, or an increase with H3K9me3 levels lead to an increase in mutation rates (Schuster-Böckler and Lehner 2012; Behjati *et al.* 2014; Blokzijl *et al.* 2016). The effect of replicating timing on mutation rate has been attributed to the depletion of free nucleotides within later replicating regions, leading to the accumulation of single-stranded DNA, and thus rendering the DNA more susceptible to endogenous DNA damage (Stamatoyannopoulos *et al.* 2009). An alternative hypothesis is that DNA mismatch repair (MMR), which is coupled with replication, is more effective in the early replicating regions of the genome; this possibility is supported by the finding that this association is not detected in the tissue of MMR-deficient patients (Supek and Lehner 2015). While on face value, it may seem surprising that replication timing is a significant determinant for the LGG samples, given that neurons are postmitotic, glial cells still retain their ability to divide and a substantial fraction of mutations detected in neuronal samples may have occurred at earlier stages in development.

### Differences between somatic tissues and testis

**Figure 3:**
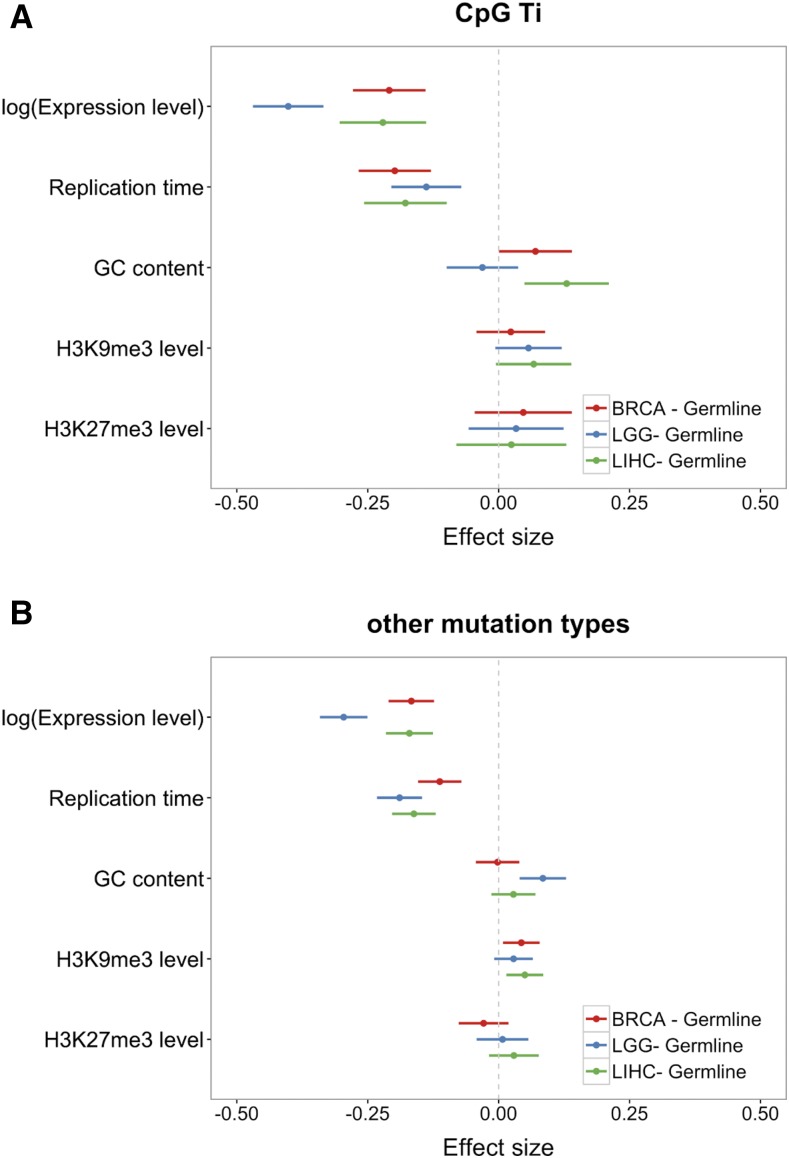
Coefficients of combined model comparing each somatic data set to germline data set using testis expression. (A) Results for CpG Ti, (B) results for other mutation types. Red, blue and green bars represent the 95% CI of the deviation of the estimated coefficient from the germline estimate; they are shown for BRCA, LGG, and LIHC data sets, respectively. For all replication timing data, a higher value means earlier.

Figure 1 and Figure 2 also hint at a difference between testis (and more tentatively, ovary) and somatic tissues in the magnitude of the effects of replication timing on mutation rates and the direction of the effects of expression levels, with a significant positive effect for germline mutations and a significantly negative effect for somatic tissues (*e.g.*, BRCA: *P* = 8 × 10^−16^ for CpG Ti; *P* < 2 × 10^−16^ for other mutation types; *P* < 2 × 10^−7^ for all somatic tissues and mutation types). When we tested for this difference explicitly, by adding a binary variable for soma and germline (see *Materials and Methods*), we found that, indeed, both expression levels and replication timing differ in their effects, for CpG Ti and other mutation types (using testis as a proxy for germline expression levels; Figure 3). The same qualitative results are obtained when using expression data from ovary instead (Figure S10 in File S1).

Notably, the effect of replication timing is stronger in soma (Figure 2 and Figure 3). The simplest explanation is that a larger fraction of mutations in the soma are introduced by errors related to replication, as opposed to other nonreplicative sources. Another (not mutually exclusive) possibility is that the effect of early replication *vs.* late replication differs to a greater extent in the soma than in the germline. For example, if MMR is much more efficient in early replicating regions (Supek and Lehner 2015) and more efficient in soma than germline.

**Figure 4:**
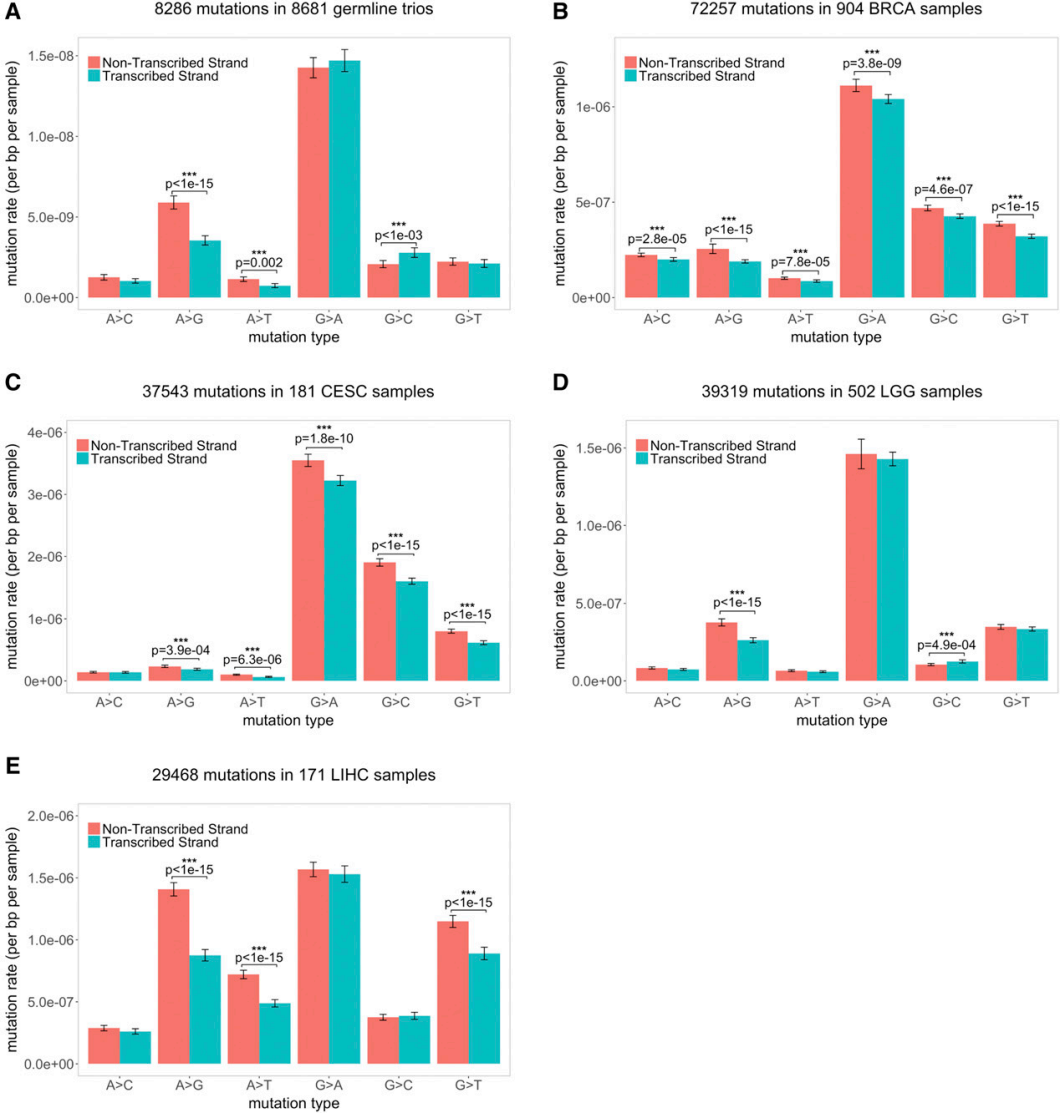
Comparison of the mutation spectrum between NTS and TS. Results for (A) germline; (B) BRCA; (C) CESC; (D) LGG; and (E) LIHC. The error bars of the mutation rate denote 95% CIs estimated by bootstrapping (see *Materials and Methods*).

In addition, there is a significant difference between the effects of expression in testis and ovary compared to all three somatic tissues considered, with a greater decrease in mutation rates with expression seen in soma (Figure 2, Figure 3, and Figure S10 in File S1). To examine this possibility further, we considered a signature of TCR—strand asymmetry—in the different tissues, detecting its presence among germline mutations as well as in all four somatic tissues (Figure 4). Consistent with previous studies (Green *et al.* 2003; McVicker and Green 2010; Francioli *et al.* 2015), one type in particular, A > G, stands out. While the asymmetry is significant (and in the same direction) in all five data sets, with more mutation on the NTS than the TS, the extent of asymmetry is significantly different among the five data sets (χ^2^ test, *P* = 3 × 10^−8^; the ratios of the mutation rates on NTS *vs.* TS are 1.66, 1.35, 1.25, 1.44, and 1.60 for germline, BRCA, CESC, LGG, and LIHC, respectively). Intriguingly, other mutation types, notably G > C mutations, show even more pronounced differences among tissues, with a significant excess on the TS in the germline and LGG samples, but a significant paucity on the NTS in BRCA and CESC. These findings indicate a potential difference in either strand-biased damage or in TCR (or both) among somatic tissues. In summary, the total mutation rate appears to behave quite differently as a function of expression levels in testis and ovary compared to soma (Figure 1, Figure 2, and Figure 3), despite the fact that we observed clear evidence for TCR in both germline and somatic mutations (Figure 4).

**Figure 5:**
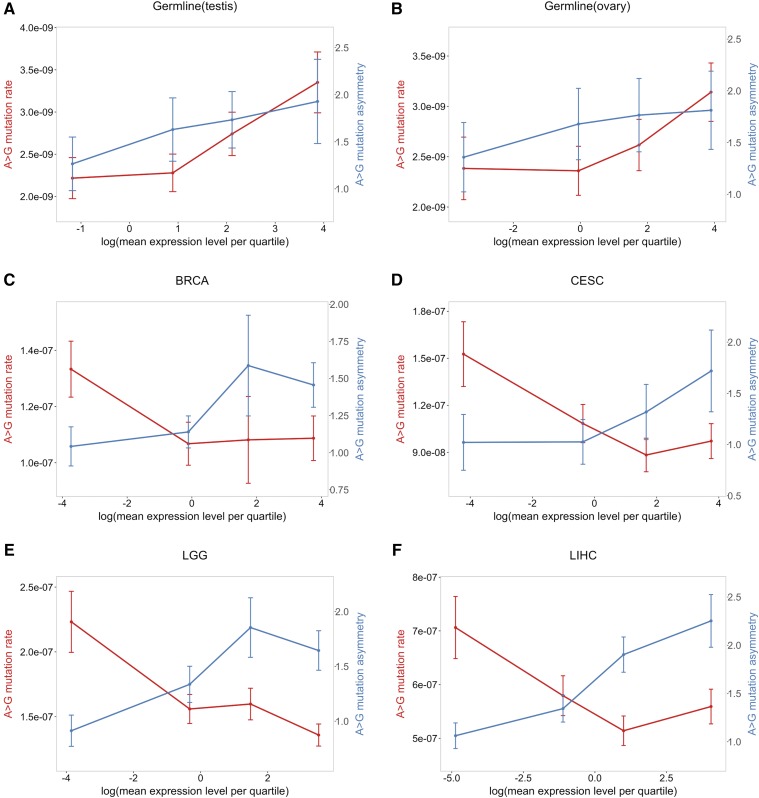
The degree of A > G strand asymmetry and the A > G mutation rate as a function of gene expression level quartiles. Shown are results for the germline using testis expression levels (A) and ovary expression levels (B); (C) BRCA; (D) CESC; (E) LGG; and (F) LIHC. The error bars for both the strand asymmetry and the mutation rate per quartile were estimated by bootstrapping (see *Materials and Methods*).

One way to visualize these differences is to focus on A > G mutations, and to consider how the mutation rate and degree of strand asymmetry vary with expression in different tissues (Figure 5). A striking contrast emerges: in testis and ovary, as expression levels increase, mutation rates and asymmetry increase, whereas, in the somatic tissues, asymmetry increases while mutation rates decrease. The same pattern is seen when A > T mutation rate and asymmetry are considered (see Figure S5 in File S1). This difference in behavior with expression levels suggests that the balance between damage and repair rates during transcription differs between germline and soma.

**Figure 6:**
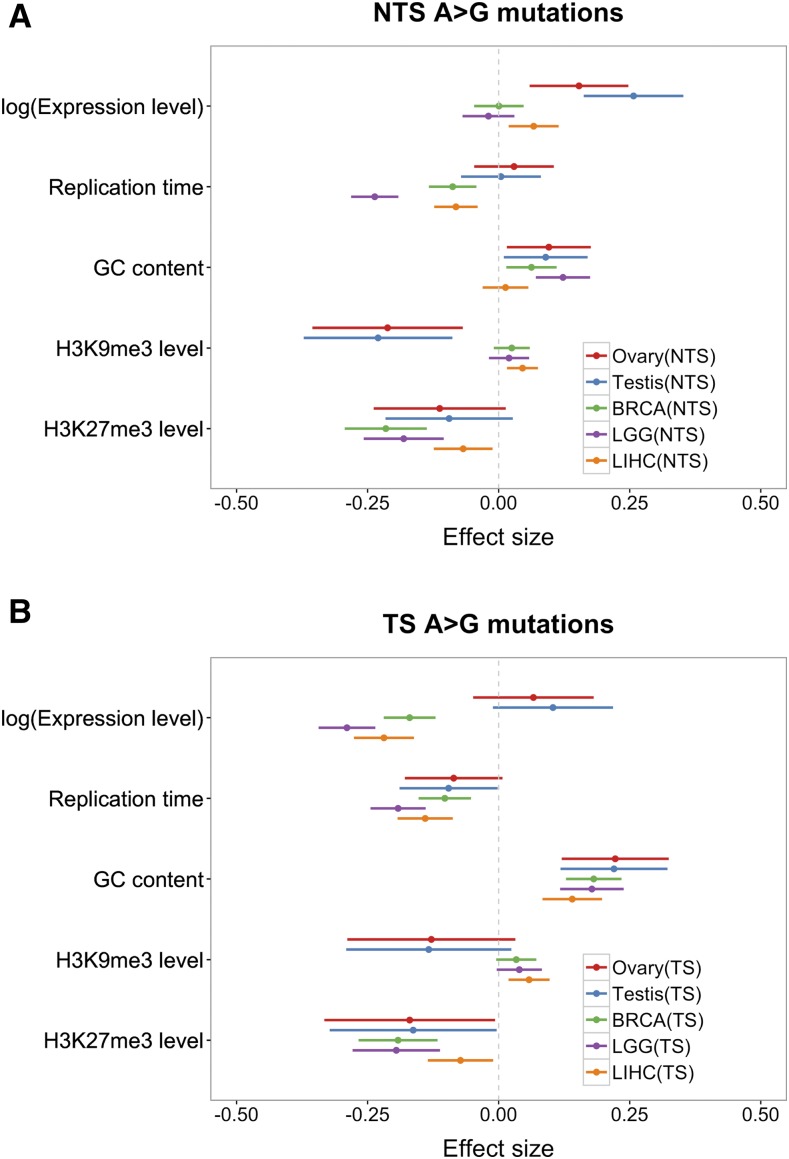
Coefficients of the multivariable binomial regression model fit to A > G mutations on NTS (A) and TS (B). Red, blue, green, purple and orange bars represent the 95% CI for the estimate of the regression coefficient in germline data set using expression levels in ovary, testis, BRCA, LGG, and LIHC. For all replication timing data, a higher value means earlier.

To explore the effects of transcription in more depth, we applied our regression model to NTS A > G mutations and TS A > G mutations separately (Figure 6). Increased expression in the soma has no discernable effect on the NTS, other than in liver, where it slightly increases mutation rates, but it decreases the mutation rate on the TS. In contrast, expression in testis and ovary leads to increased mutation rates on the NTS, and little or no elevation on the TS. Assuming there is no repair of the NTS by TCR, these findings indicate that transcription in the germline introduces greater damage than it does in the soma, and, in both cases, that damage is efficiently repaired on the TS strand. If, however, the NTS is occasionally repaired by TCR or some other mechanism, then the findings indicate that the efficiency of TCR (relative to the damage rate) is greater in soma. In this regard, we note that when the same analysis is applied to A > T mutations, which show significant asymmetry in all tissues considered other than LGG, there is some evidence that mutation rates decrease with expression in somatic tissues even on the NTS, suggesting some form of repair of the NTS coupled to transcription (Figure S6 in File S1).

## Discussion

We compared the determinants of mutation in the soma and the germline, using the same unit of analysis (a coding region) and the same statistical model, and applied it to similar exome data for germline *de novo* mutations and four types of tumors, in which mutations largely predate tumorigenesis. We recapitulated previous findings of the effects of GC content and of a histone mark indicative of repression on germline and somatic mutations, as well as those of expression levels and replication timing on somatic mutations (Schuster-Böckler and Lehner 2012; Lawrence *et al.* 2013). Strikingly, we also found clear differences in the determinants of mutation rates between germline and soma, consistent with earlier hints based on divergence data (Hodgkinson and Eyre-Walker 2011). Notably, our results confirmed that somatic mutation rates decrease with expression levels, and reveal that, in sharp contrast, *de novo* germline mutation rates increase with expression levels in testis (and more tentatively, in ovary). This contrast suggests that transcription may be mutagenic in germline cells but not in soma, and that the DNA damage or repair processes differ between them.

One limitation of our comparison—and of previous studies of germline and somatic mutation—is the need to rely on proxies for determinants of interest, such as replication timing data from cancer cell lines instead of normal cells and, perhaps most importantly, the use of testis and ovary as a proxy for germ cells. One difficulty in that regard is that so-called “germline mutations” actually arise from many stages of development, including cell types that predate the specification of the germline (see, *e.g.*, Rahbari *et al.* 2016) and thus it is difficult to know which of the available tissues to use as a proxy. Until these findings can be revisited with expression data from more precise cell types, such as primordial germ cells and spermatocytes, all that can be concluded is that our findings point to a difference between somatic tissues and some subset of germ cells.

A second limitation is that we considered only two types of mutations (CpG Ti and other). While these two types capture most of the variation in mutation rates, the larger context (adjacent base pairs, but also 7mers) also impacts mutation rates (Hwang and Green 2004; Hodgkinson and Eyre-Walker 2011; Aggarwala and Voight 2016). These different mutation subtypes are likely affected somewhat differently by the determinants considered here (Carlson *et al.* 2017). Despite these limitations, our work provides a framework to contrast possible determinants of mutation rates in soma and germline while controlling for some confounding effects, and results will only improve as data sets increase and the measurements of salient genomic and cellular features become more accurate.

What is already clear is that there exist divergent effects of expression on mutation rates across tissues that are not attributable to well-known covariates. Moreover, the differences cannot readily be explained by the noise introduced by imperfect proxies or limited data. One possibility is that the effects of transcription do not vary across tissues, but are nonlinear in their effects on mutation rates. As a thought experiment, if genes that are not expressed were not repaired, and had a relatively high mutation rate as a result, and genes that are highly expressed had a high mutation rate because the repair efficiency is insufficient relative to damage, then genes with low levels of expression would be the least mutagenic. If so, tissues in which many genes have either low or no expression might show a decrease of mutation rates with expression, whereas tissues with many genes that are lowly or expressed might show an increase in mutation rates with expression.

A more likely explanation, in our view, is that the tradeoff between damage and repair associated with transcription differs among tissues, and in particular between germline and soma. Indeed, we know that tissue differs by sources of damage (Alexandrov *et al.* 2015) and the rate at which mutations accumulate (Blokzijl *et al.* 2016). There also exist differences in the signatures of strand asymmetry (Alexandrov *et al.* 2013; Blokzijl *et al.* 2016; Figure 4). Transcription plausibly increases the rate of damage by opening up the DNA helix, rendering the single strands more susceptible to mutagens (Polak and Arndt 2008; Jinks-Robertson and Bhagwat 2014). One possibility is that, in the germline, transcription-associated mutagenesis (TAM) swamps TCR, leading to higher mutation rates with increased transcription, whereas in the soma, TCR is more efficient, especially on the TS, and the balance of TAM and TCR leads to decreased mutagenesis with increased expression. Another possibility, which is not mutually exclusive, is the presence of additional repair mechanisms in somatic tissues. In support of this possibility, global genome repair (GGR) is attenuated in differentiated cells, yet mutations on the NTS appear to nonetheless be repaired efficiently (Nouspikel and Hanawalt 2000; Marteijn *et al.* 2014). This evidence led to the hypothesis of transcription-domain-associated repair (DAR), which might repair damage on both strands in addition to TCR (reviewed in Nouspikel 2007). From an evolutionary standpoint, the increased efficiency of TCR relative to TAM in soma *vs.* germline may be explained by selection pressure on the repair of somatic tissues to prevent aging and cancer (Lynch 2010).

Mounting evidence suggests that per cell division mutation rates differ across tissues (Greenman *et al.* 2007; Lynch 2010; Alexandrov *et al.* 2013), and, in particular, that they may be higher in early embryonic development than at other stages of development (Ségurel *et al.* 2014; Harland *et al.* 2016; Lindsay *et al.* 2016; Rahbari *et al.* 2016). This study raises the possibility that at least part of the explanation may lie in the balance between damage and repair, with TCR operating at different efficiencies relative to TAM or jointly with other repair pathways, thereby maintaining low mutation rates in soma. As mutation data from more tissues become available, it will be both feasible and enlightening to examine tissue-specific differences in repair.

## Supplementary Material

Supplemental material is available online at www.genetics.org/lookup/suppl/doi:10.1534/genetics.117.1114/-/DC1.
