# Supplementary material for: Contrasting Determinants of Mutation Rates in Germline and Soma
Source: Genetics. 2017 Jul 21;207(1):255–67. doi: 10.1534/genetics.117.1114 (PMC5586376; doi:10.1534/genetics.117.1114)
Supplement: Supplementary file 1 [file 255FileS1.pdf]

Supplementary Figures

A.

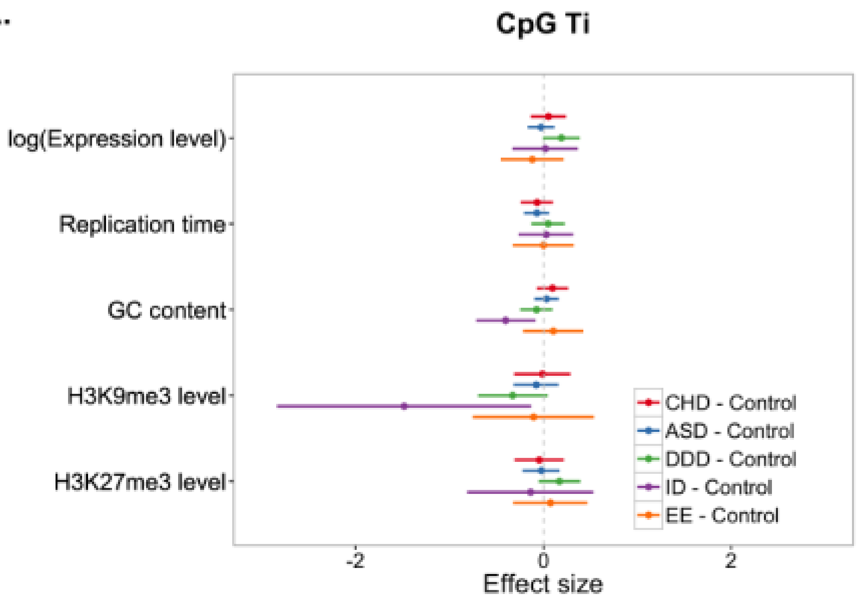

B.

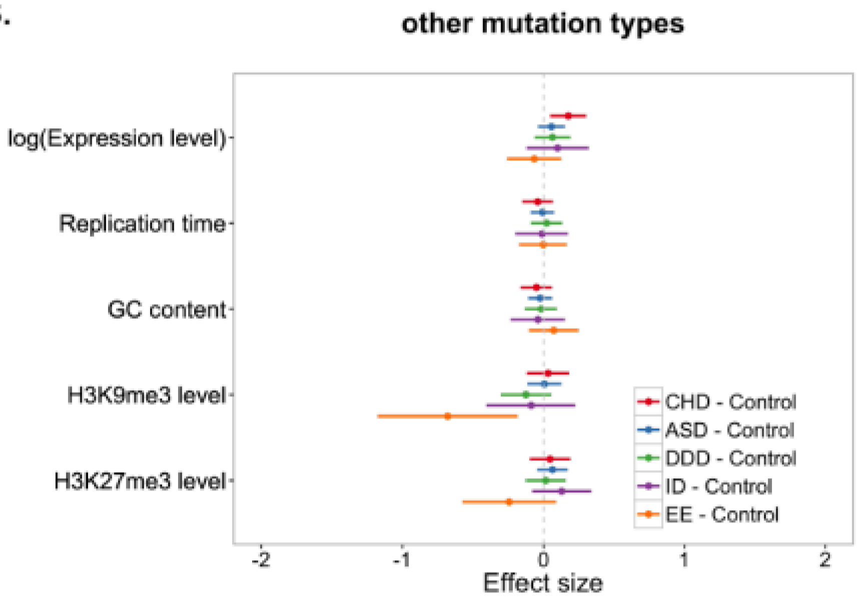

Figure S1. Coefficients of model testing for heterogeneity in the determinants of mutation rates among germline datasets. In panel A are results for CpG Ti and in panel B for other mutation types. Red, blue and green bars represent the 95% CI of the deviation of the estimated coefficient from that in the control (unaffected) group, shown for congenital heart defect (CHD), autism spectrum disorder (ASD), deciphering developmental disorder (DDD) datasets, intellectual disability (ID) and Epileptic Encephalopathies (EE), respectively. For all replication time data, a higher value means earlier.

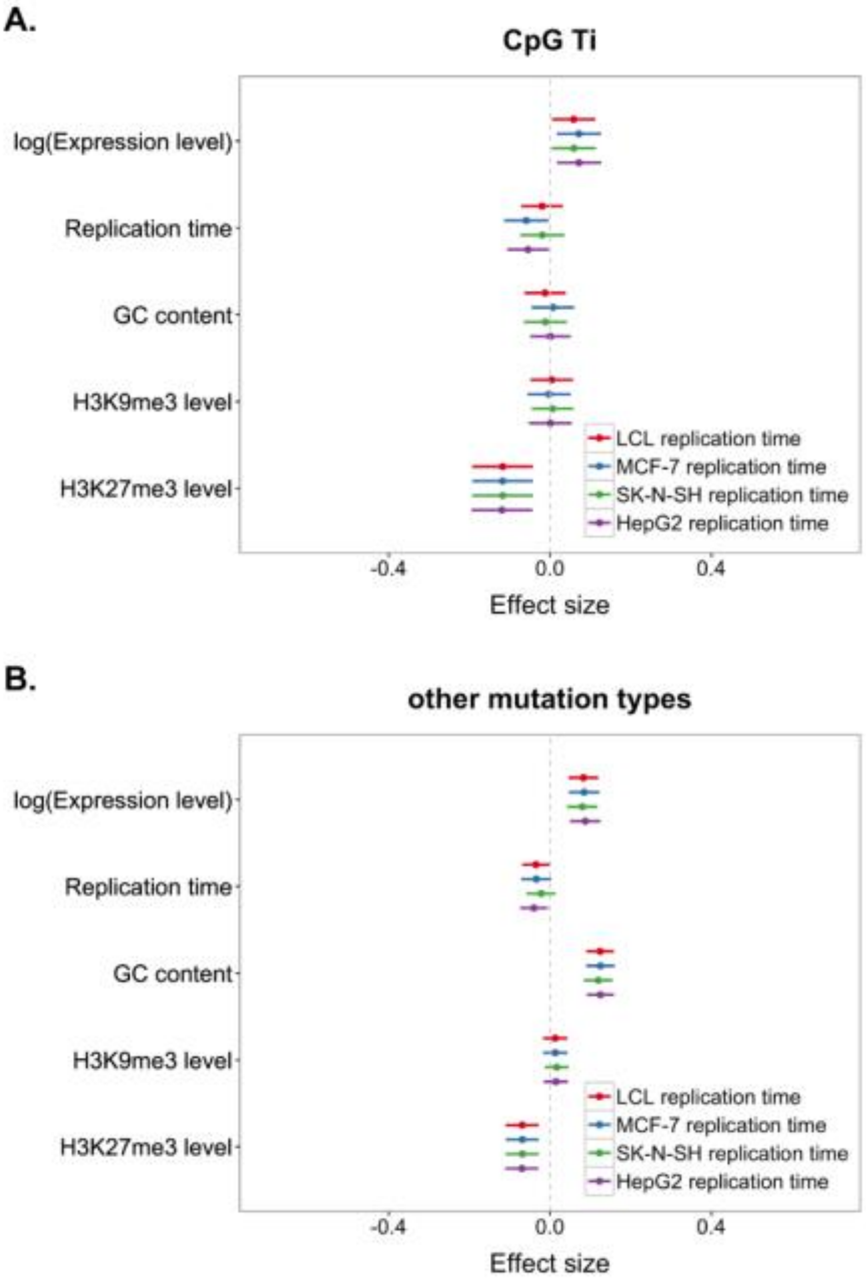

Figure S2. Comparing models with (blue) and without interaction terms (red). Panels A to H report results for CpG Ti and other mutation types in germline, BRCA (breast invasive carcinoma), LGG (brain lower grade glioma) and LIHC (liver hepatocellular carcinoma) data sets, respectively. For all replication time data, a higher value means earlier. Bars denote 95% CI for the estimate of the regression coefficient.

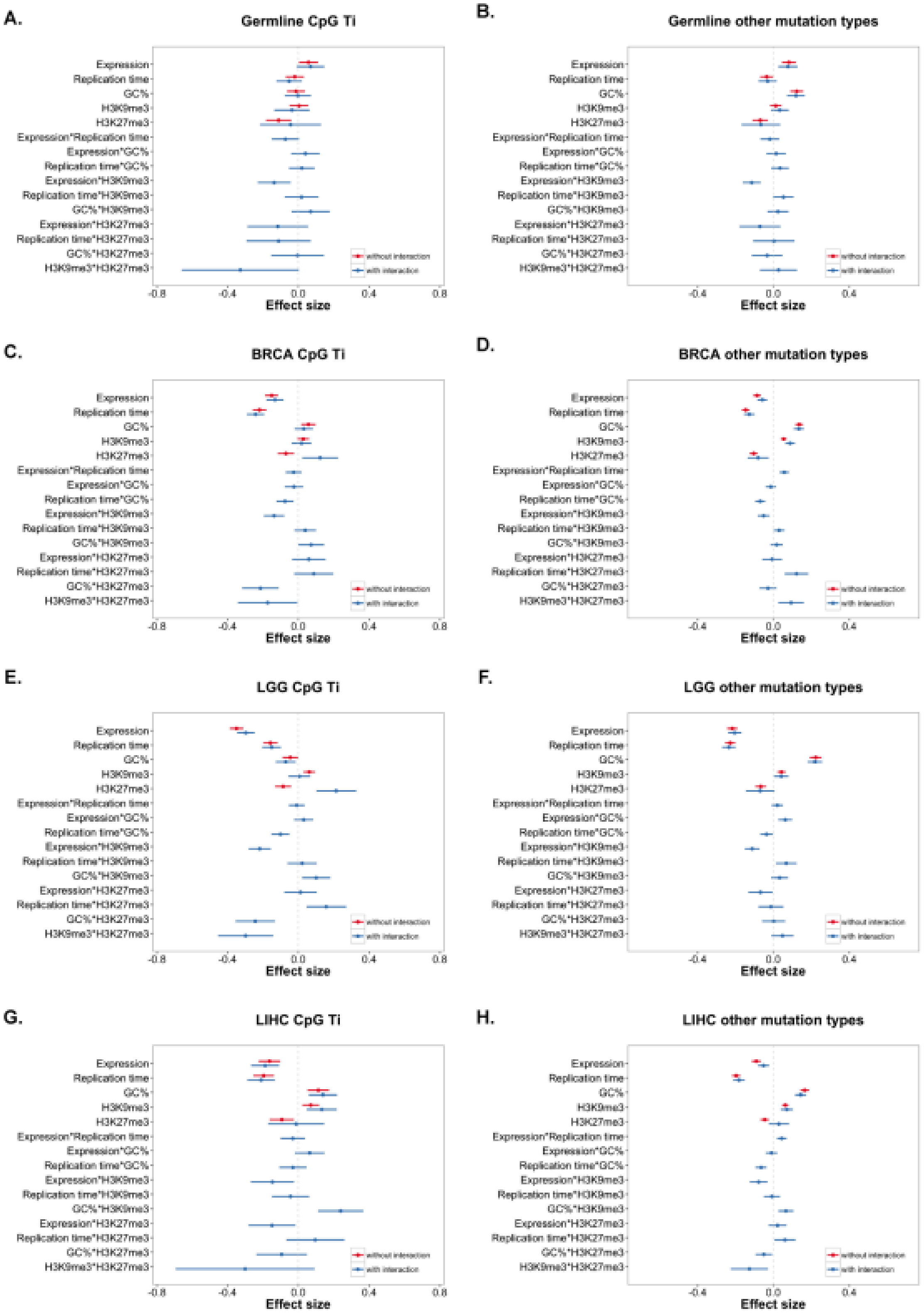

Figure S3. Comparing models with (blue) and without interaction terms (red). Panels A to H report results for CpG Ti and other mutation types in germline, BRCA (breast

invasive carcinoma), LGG (brain lower grade glioma) and LIHC (liver hepatocellular carcinoma) data sets, respectively. For all replication time data, a higher value means earlier. Bars denote 95% CI for the estimate of the regression coefficient.

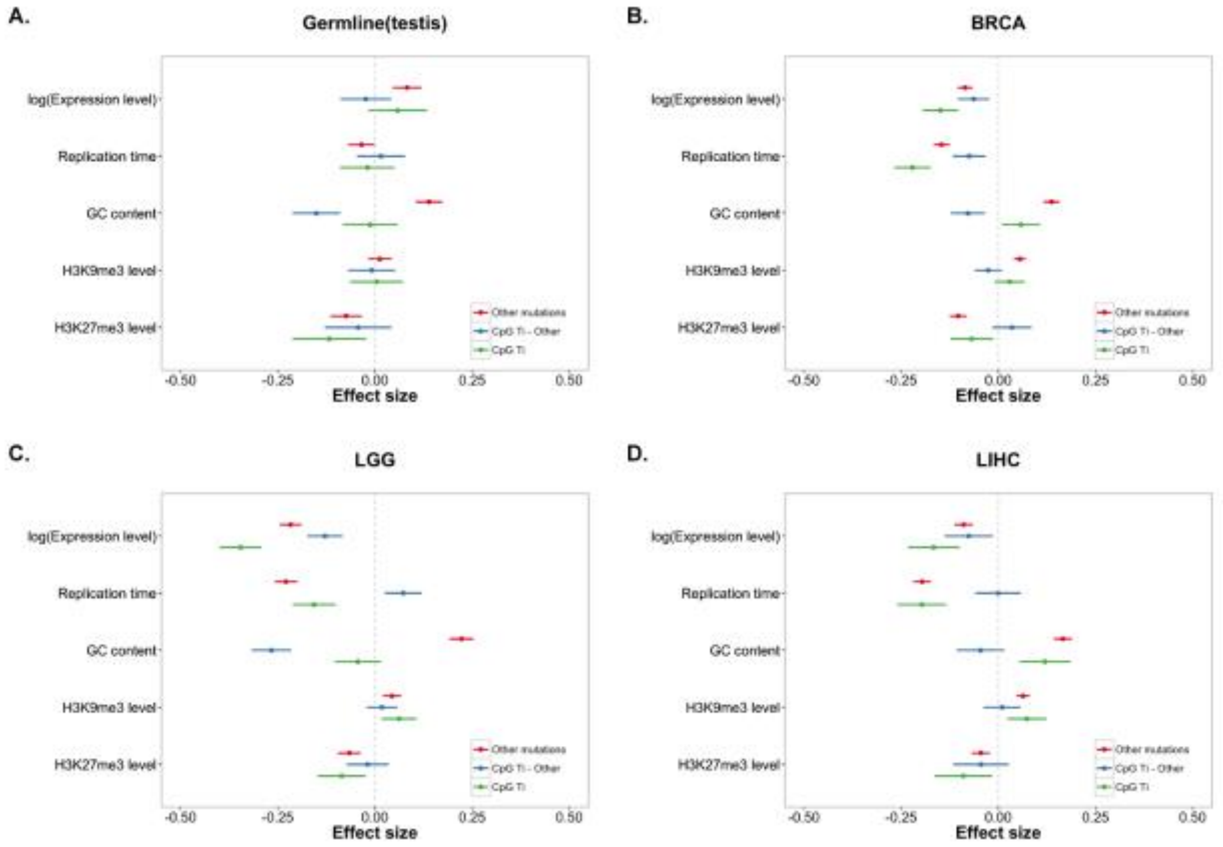

Figure S4. Regression coefficients from the combined model of CpG Ti and other mutations. In panel A are results for the germline using testis expression; in panel B, for breast tissue; in panel C, for brain tissue; and in panel D, for liver tissue. Black, blue and red bars represent coefficients for other mutations, coefficients for CpG Ti and the deviation of the estimated coefficient in CpG Ti from other mutations, respectively. Bars represent 95% CI for the estimate of the regression coefficient. For all replication timing data, a higher value means earlier.

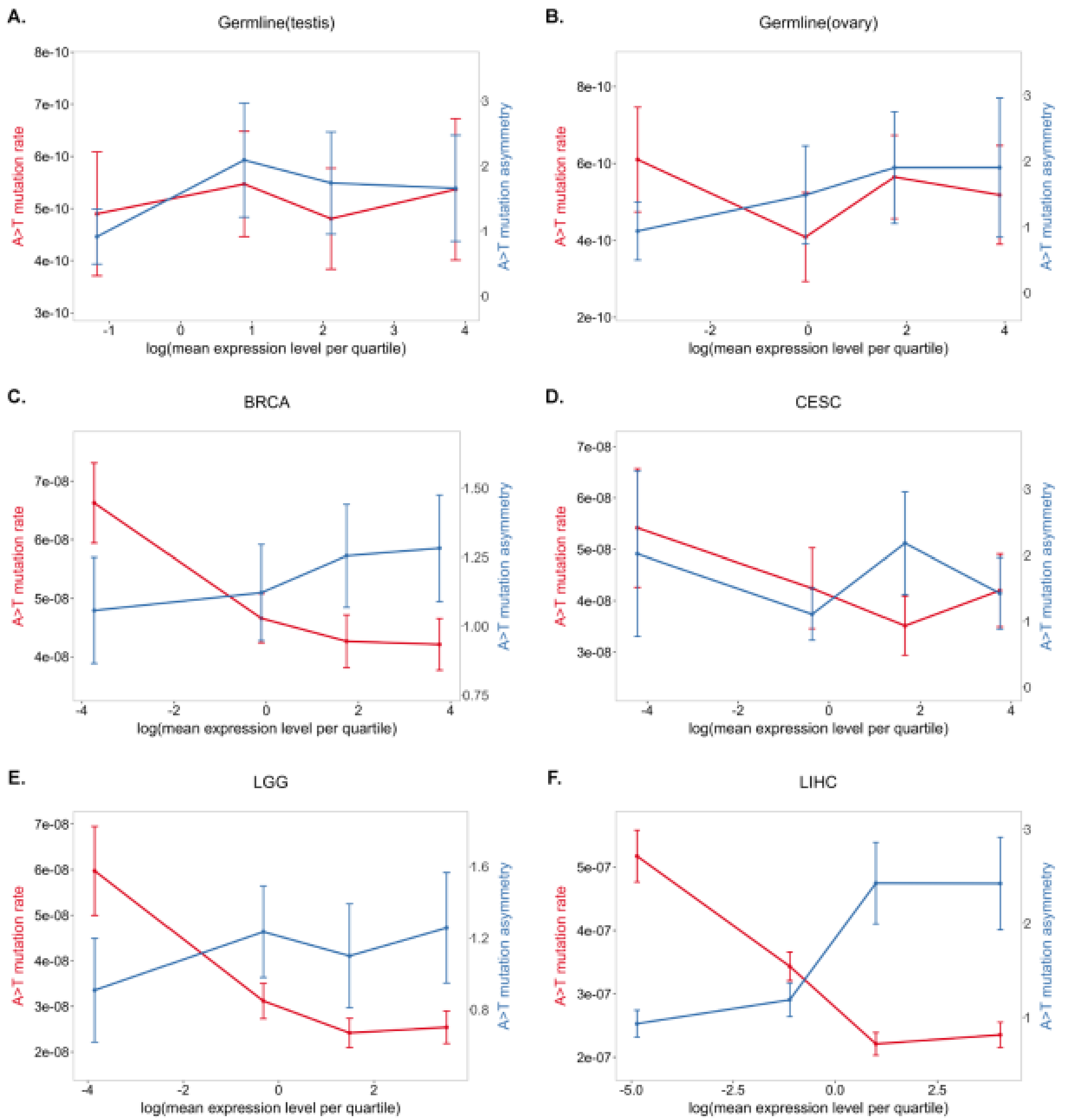

Figure S5. The degree of A>T strand asymmetry and the A>T mutation rate as a function of gene expression level quartiles. Shown are in panels A and B are results for the germline using testis expression levels and ovary expression levels, respectively; in panel C, for BRCA (breast invasive carcinoma); in panel D, for CESC (cervical squamous cell carcinoma and endocervical adenocarcinoma); in panel E, for LGG (brain lower grade glioma); and in panel F, for LIHC (liver hepatocellular carcinoma). The error bars for both the strand asymmetry and the mutation rate per quartile were estimated by bootstrapping (see Materials and Methods).

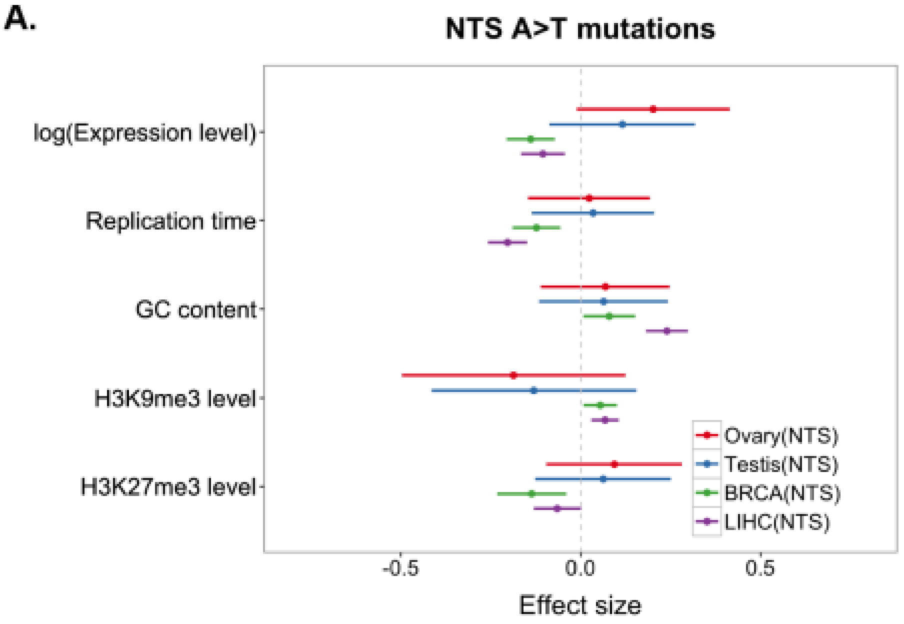

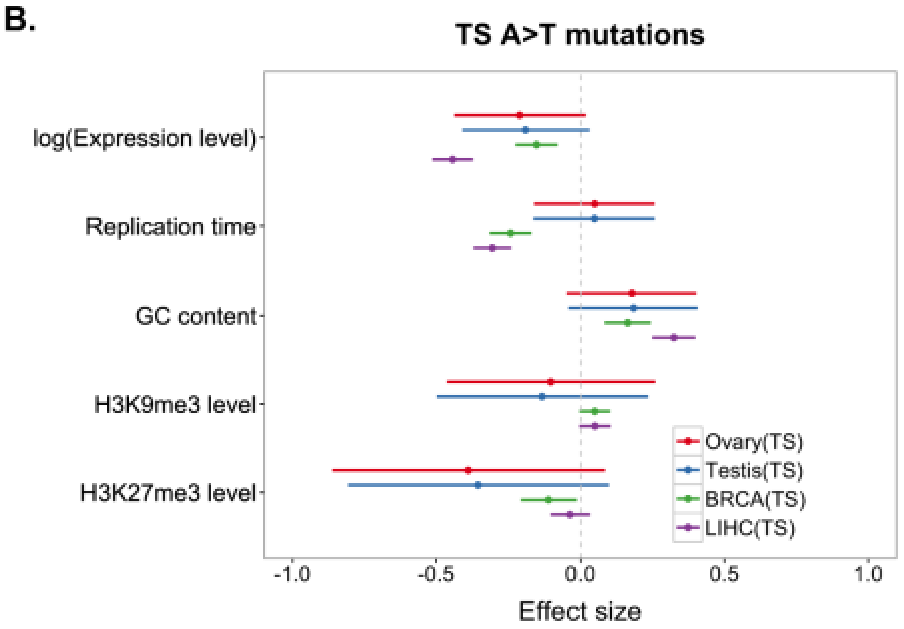

Figure S6. Coefficients of the multivariable binomial regression model fit to A>T mutations on NTS (panel A) and TS (panel B). Red, blue, green, purple and orange bars represent the 95% CI for the estimate of the regression coefficient in germline data set using expression levels in ovary, testis, BRCA (breast invasive carcinoma), LGG (brain lower grade glioma) and LIHC (liver hepatocellular carcinoma). For all replication timing data, a higher value means earlier.

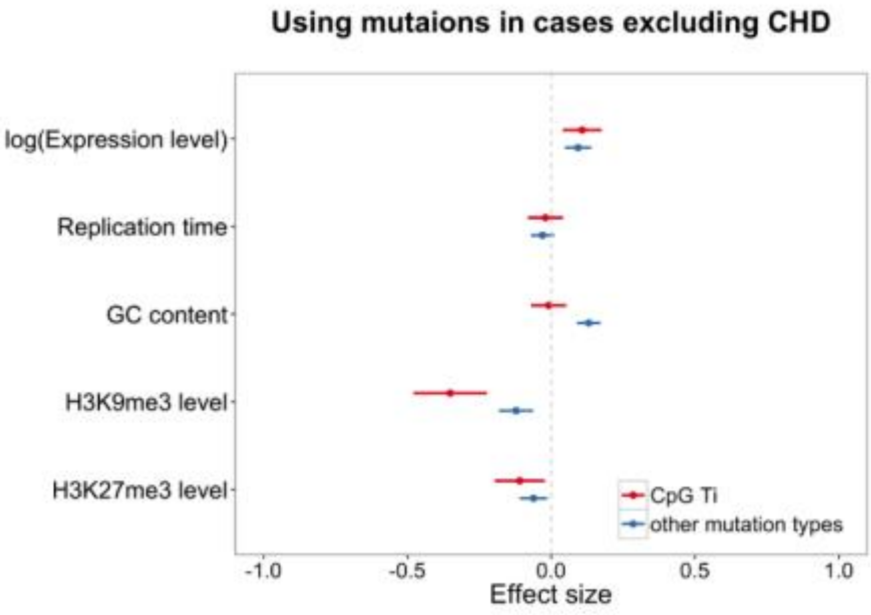

Figure S7. Coefficients of multivariable binomial regression model fit to germline mutation in four sets of cases excluding CHD. Red and blue bars represent the 95% CI for the estimate of the regression coefficient in CpG Ti and other mutation types, respectively. For all replication timing data, a higher value means earlier.

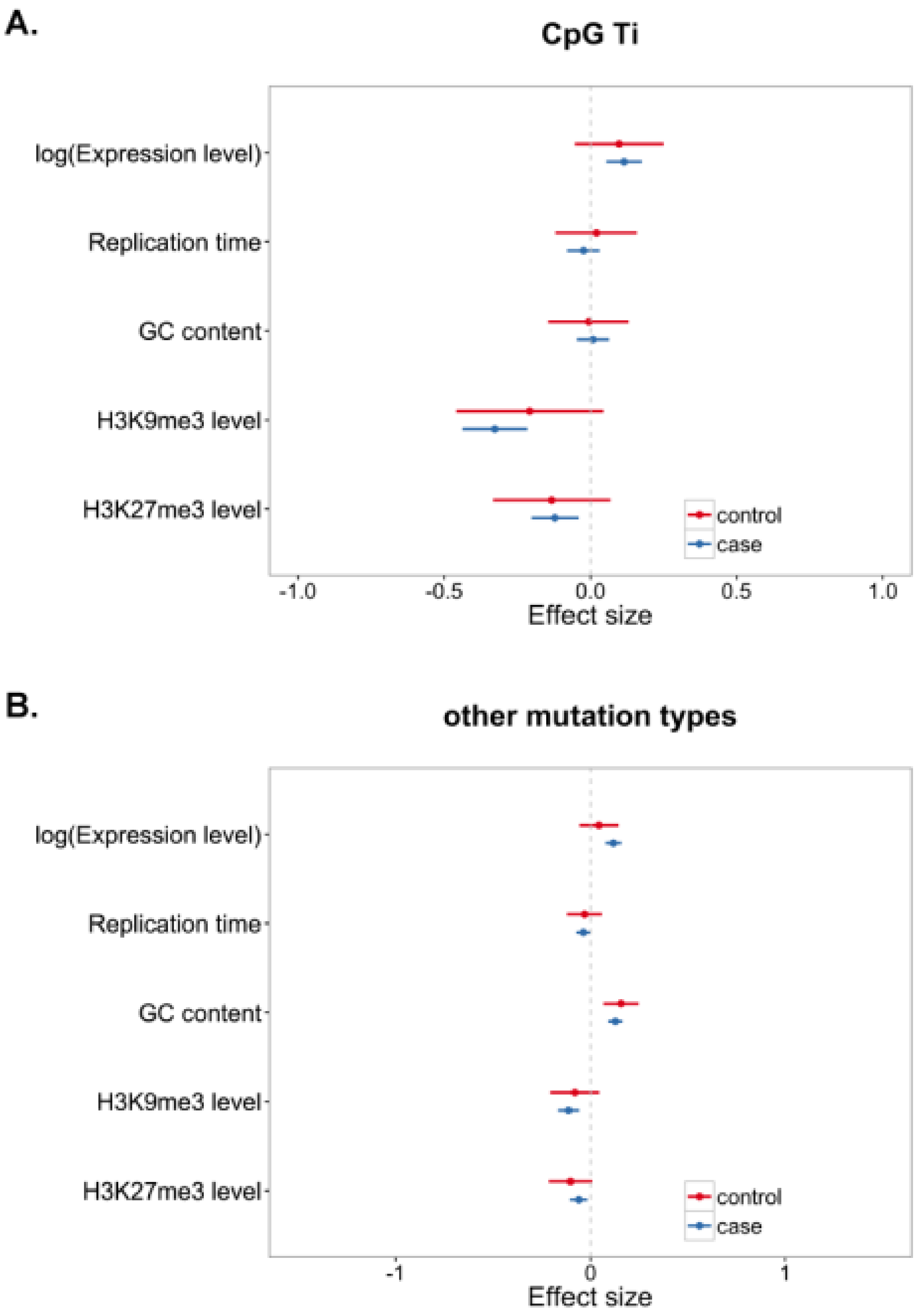

Figure S8. Coefficients of model testing for heterogeneity in the determinants of mutation rates between cases and controls. In panel A are results for CpG Ti and in panel B for other mutation types. Red and blue bars represent the 95% CI of the estimated coefficient in the control (unaffected) group and all cases combined (ASD+CHD+DDD+EE+ID), respectively. For all replication timing data, a higher value means earlier.

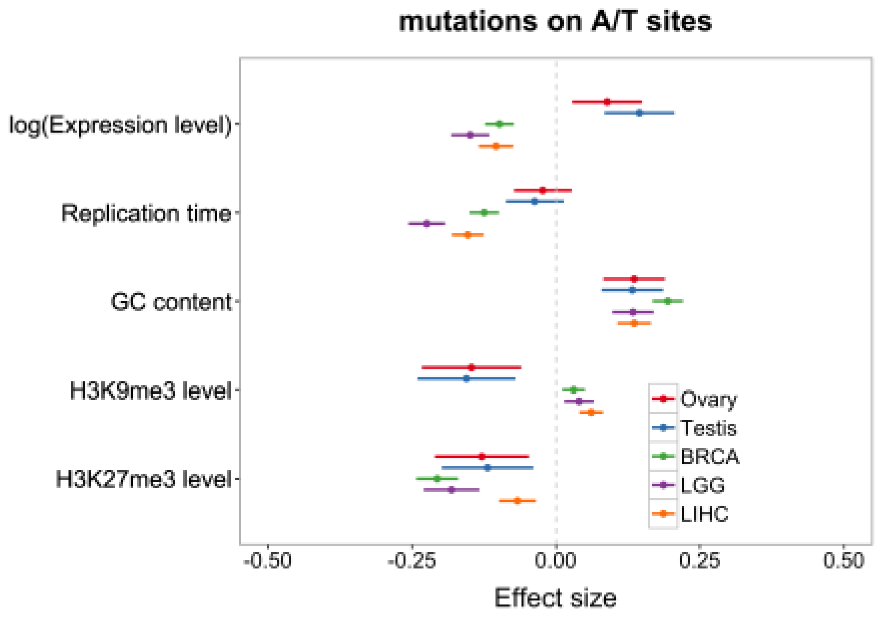

Figure S9. Coefficients of model fit to mutations at A or T base pairs. Red, blue, green, purple and orange bars represent the 95% CI of the regression coefficient using ovary expression and testis expression to predict germline mutation rates and BRCA (breast invasive carcinoma), LGG (brain lower grade glioma) and LIHC (liver hepatocellular carcinoma) to predict somatic mutation rates, respectively. For all replication timing data, a higher value means earlier. The model used here is the same as in Figure 1, but the offset term L is the number of A or T base pairs, rather than all base pairs.

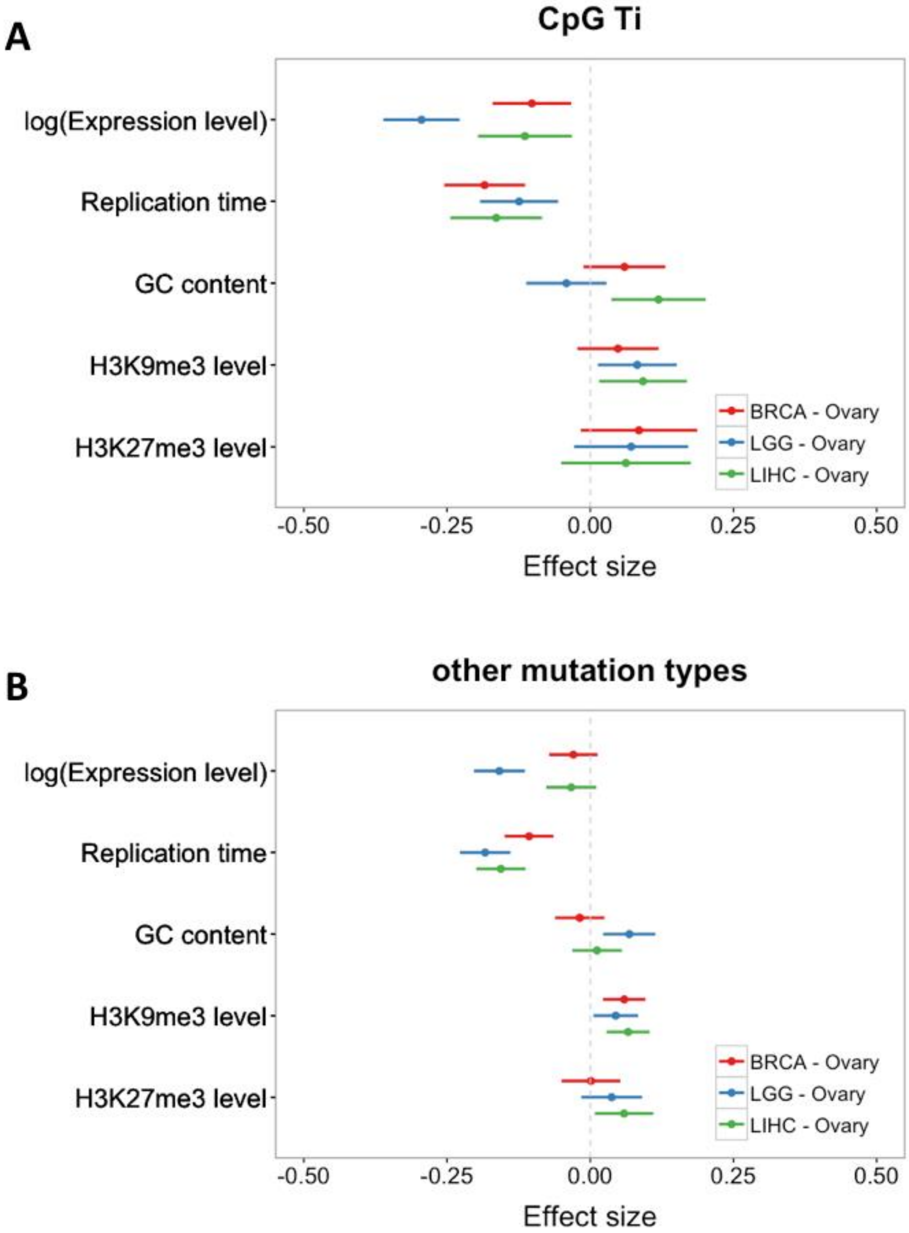

Figure S10. Coefficients of combined model comparing each somatic data set to germline data set using ovary expression. In panel A, results for CpG Ti and in panel B, for other mutation types. Red, blue and green bars represent the 95% CI of the deviation of the estimated coefficient from the germline estimate; they are shown for BRCA (breast invasive carcinoma), LGG (brain lower grade glioma) and LIHC (liver hepatocellular carcinoma) data sets respectively. For all replication timing data, a higher value means earlier.
